# Prevention or Cure

**Published:** 1908-06-27

**Authors:** 


### The Hospital

A Journal of

The Medical Sciences and Hospital Administration.

New Series. No. 68, Vol. III. [No. 1140, Vol. XLIV.] SATURDAY, JUNE 27, 1908.

### Prevention or Cure.

We do not know the age of the adage which has suggested the heading to these lines, but it is fair to credit it with a respectable antiquity, for its gist is platitudinous enough. Yet, like many another sage proverb, it has, until comparatively lately, had but little influence upon practice. This at least is the reflection which rises to the mind upon a survey of the history of preventive medicine. But it is not literally true, for even in the old mystical days, when medicine and superstition were almost to be considered interchangeable terms, when pestilence was reckoned a manifestation of malignity upon the part of a supernatural power, prevention was certainly attempted. It is hard to look upon charms and exorcisms as items of preventive medicine, but we must regard these efforts as the lineal ancestors of the isolation hospital, the cult of fresh air, and all the other elements of latter-day hygiene. Still, in spite of the technical fallacy, preventive medicine is, as we have said, of comparatively recent growth, and, extraordinary as have been the benefits which have accrued from it, we cannot escape the conclusion that they have fallen far short of the ideal. It is true that plague, small-pox and typhus fever, the prominent scourges of mediæval Europe, have practically ceased to exist among us. But what are we to think of a civilisation which, after so many years of knowledge, still year by year in this kingdom allows more than one hundred thousand infants to die in their first year, mostly from preventable diseases, while a preventable malady, pulmonary tuberculosis, continues to claim its victims in similar numbers? Why is it that when knowledge is the only thing needful for the saving of life we yet find the dissemination of this vital safeguard so little accounted of? The answer has been given, in another connection, by Lord Justice Fletcher Moulton. In his evidence, recently given before the Royal Commission on Vivisection, he spoke as follows: "If they were to ask a man who had given thought to the matter, who of all living men had been privileged to do most to relieve pain, the answer would almost without exception be, Lord Lister. . . . Yet he saw the other day that at an anti-vivisection meeting Lord Lister's name was mentioned, and it was greeted with shouts of 'brute.' That was perfectly consistent. . . . The prevented pain, which had made Lord Lister's name so esteemed, was not present to their minds. The inflicted pain struck their imagination." Herein lies the true explanation of the lagging steps of preventive medicine. Present suffering touches the imagination. The wasted cheeks of the consumptive, the skeleton frame of the infant worn with chronic gastro-enteritis, these are tangible. They quicken interest, command attention, soften hearts and open purses, and the consequence is that all over the country there have arisen hospitals and sanatoria for the alleviation of the suffering individual. But prevention is a dull business. Mankind in bulk has a wonderful command of cheery optimism which enables it to dismiss from among its preoccupations all possibilities of discomfort which are even tolerably remote; and it appears to be almost hopeless to awaken a living interest in the prevention of those calamities whose cure—a far harder task—is pursued with unceasing solicitude.

Very little contemplation is required to show the Gilbertian nature of such a system, and the subject is much too solemn to be allowed to rest in this most unsatisfactory condition. However laborious the task, those among us who are really concerned for the health and happiness of our fellows must continue to urge the relative futility of attacking the problem from the wrong end. We do not mean to suggest the diversion to purposes of hygienic education of funds appropriated to the service of suffering individuals, although on academic grounds there is much to be said for the proposition. It is a feature of progressive civilisation that the claims of the individual tend to become more and more paramount at the expense of posterity and the welfare of the race. But although it may not be possible to concentrate our hygienic efforts upon prevention alone, there is no reason why both movements should not be made to advance side by side, and at a greater speed than in the past. Already there has been progress, and there is no doubt that the declining mortality from pulmonary tuberculosis in England which has been witnessed in the last few years, is largely due to the spread of knowledge concerning the methods by which the disease is diffused. But in Ireland the contrary has obtained, and the ravages of consumption have been steadily increasing. We confidently expect, however, that the movement inaugurated last year by Lady Aberdeen will remedy this growing mischief and bear the fruits it deserves, for it is plainly the scientific and rational method. She has started a society called "The Woman's National Health Association of Ireland," which has in view a threefold object. Firstly, to reduce the mortality from consumption; secondly, to reduce infant mortality in the larger towns; and thirdly, to improve the hygienic condition of the national schools. It would seem that the measures adopted are precisely of the kind calculated to develop what is, after all, but a specialised type of the instinct of self-preservation. A tuberculosis exhibition was organised, which not only demonstrated the different forms of the disease, but supplied lecturers to explain its methods of dissemination, and the principles underlying its prevention. This exhibition was so successful that it became necessary to duplicate it, one body of representatives touring the north of Ireland and the other the south. How ready the people were to welcome the gospel of hygiene is proved by the fact that up to the present some 700,000 people, representing one-sixth of the population, have visited these exhibitions. Lady Aberdeen is much to be congratulated upon her public spirit and foresight. She has undertaken an arduous task and one whose rewards are gathered slowly; but they will come to her and her assistants as surely as the night follows the day. May she find many imitators!

